# Hepatocyte growth factor‐modified adipose‐derived mesenchymal stem cells inhibit human hypertrophic scar fibroblast activation

**DOI:** 10.1111/jocd.16509

**Published:** 2024-08-18

**Authors:** Tianli Zhang

**Affiliations:** ^1^ Medical College Wuhan University of Science and Technology Wuhan Hubei China

**Keywords:** adipose‐derived mesenchymal stem cells, hepatocyte growth factor, hypertrophic scar fibroblasts, nucleoside‐modified messenger RNA

## Abstract

**Purpose:**

Nucleoside‐modified messenger RNA (modRNA) holds the potential for facilitating genetic enhancement of stem cells. In this study, modRNA encoding hepatocyte growth factor (modHGF) was used to chemically modify adipose‐derived mesenchymal stem cells (ADSCs) and the effect of modified ADSCs on the activation of hypertrophic scar fibroblasts (HSFs) was evaluated.

**Methods:**

CCK‐8, wound healing, and transwell assays were utilized to evaluate the viability and migratory potential of modHGF‐engineered ADSCs and their effect on HSF activation. Reverse transcription‐polymerase chain reaction, western blot, and immunofluorescence staining were performed to detect the expression of collagen‐I (Col I), collagen‐III (Col III), alpha‐smooth muscle actin (α‐SMA), matrix metallopeptidase 1 (MMP‐1), and MMP‐3.

**Results:**

Transfection of ADSCs with modHGF (HGF‐ADSC) resulted in enhanced production of HGF. Meanwhile, modHGF modification enhanced the viability and migration of ADSCs. Notably, culture media from HGF‐ADSCs exhibited a more potent inhibitory effect on the proliferation and migration of HSFs. In addition, culture media from HGF‐ADSCs inhibited extracellular matrix synthesis of HSFs, as evidenced by reduced expression levels of Col I, Col III, and α‐SMA, while increasing expression of MMP‐1 and MMP‐3. Conversely, neutralization experiments confirmed that these effects could be effectively alleviated by blocking HGF activity.

**Conclusion:**

modHGF modification optimizes the inhibitory effect of ADSCs on HSF activation, which provides a promising alternative for preventing and treating hyperplastic scars.

## 1 BACKGROUND

Hyperplastic scar (HS) is a common fibroplastic disorder in plastic surgery, with an incidence of up to 70% among burn patients.^1^ The main features of HS are dermal thickening, protruding from surrounding healthy skin, cicatricial contracture, and pruritus, which seriously affects the appearance and physical functionality of patients while causing serious psychological problems.^2^ Numerous studies have demonstrated that the abnormal activation of fibroblasts facilitates the pathological progression of HS.^3^, ^4^ Following cutaneous injury, activated fibroblasts proliferate and migrate, and secrete abundant type I collagen (Col I), type III collagen (Col III), type IV collagen (Col IV), and α‐smooth muscle actin (α‐SMA). This excessive secretion leads to extracellular matrix deposition which further exacerbates HS formation.^4^, ^5^ Therefore, it is essential to prevent hypertrophic scar fibroblast (HSF) activation and eliminate extracellular matrix excessive deposition for effective HS treatment.

Adipose‐derived mesenchymal stem cells (ADSCs) are a kind of mesenchymal stem cells capable of multidirection differentiation. ADSCs possess immunomodulatory, anti‐fibrosis, and angiogenesis promotion.^6^, ^7^, ^8^ In recent years, ADSC transplantation has emerged as an innovative therapeutic approach for HS, facilitating tissue repair, promoting wound healing, and reducing scar formation via homing and/or paracrine mechanisms.^9^, ^10^, ^11^ However, conventional ADSC‐based therapies for various scars have not achieved satisfactory clinical outcomes. Recently, nucleoside‐modified messenger RNA (modRNA) technology represents an alternative strategy for transiently inducing gene expression.^12^, ^13^ The modRNA molecules encoding multiple pluripotent factors have been demonstrated to improve the therapeutic effect of ADSCs.^13^ Several studies have indicated that insulin‐like growth factor 1 (IGF‐1) modRNA‐engineered ADSCs significantly ameliorate osteoarthritis development and corneal injury.^14^, ^15^ Another study showed that VEGF modRNA‐modified ADSCs prominently improved fat graft survival 1–3 months after in vivo transplantation.^16^ Therefore, modRNA engineered‐ADSCs hold promise for the treatment of HS.

Hepatocyte growth factor (HGF), a multifunctional tissue growth factor, is regarded as one of the most potent anti‐fibrotic and anti‐apoptotic factors.^17^ A report confirmed that HGF could effectively inhibit the activation of HSFs and antagonize tissue fibrosis in vivo,^18^ highlighting the anti‐fibrotic role of HGF. In a recent study, HGF modRNA (modHGF)‐modified human umbilical cord MSCs have been found to enhance ovarian function in chemotherapy‐induced premature ovarian insufficiency rats via promoting angiogenesis and inhibiting fibrosis,^19^ This indicates that HGF exerts a crucial role of MSCs against fibrosis. In addition, HGF exhibits mitogenic, mitogenic, and morphogenic properties across various cell types, including MSCs. Wang M et al.^20^ showed that HGF enhances the viability and mobility of deer antler MSCs. However, the potential efficacy of modHGF‐engineered ADSCs in HS has rarely been reported.

In this study, we postulated that modHGF‐engineered ADSCs could inhibit the activation of HSFs. To investigate this hypothesis, we initially assessed the effect of modHGF on the cellular behaviors of ADSCs. Subsequently, we examined whether modHGF‐engineered ADSCs could alter the proliferation and migration of HSFs. Additionally, we investigated the influence of modHGF‐engineered ADSCs on the extracellular matrix synthesis in HSFs.

## 2 METHODS

### 2.1 Isolated and culture of human ADSCs and HSFs

Human ADSCs were isolated from human abdominal subcutaneous adipose tissues and cultured in a modified MEM with 10% FBS. HSFs were isolated from hypertrophic scar tissue samples as previously reported. The tissue was cut into small pieces and digested with 0.3% trypsin/PBS at 4°C for 10 h, followed by the addition of DMEM (Gibco, USA). The tissue pieces were left to dry in the incubator for 4–6 h and then maintained in DMEM with 10% FBS at 37°C under 5% CO_2_. When the single‐layer fibroblasts began to fuse and form dense cell colonies, they were passaged at a ratio of 1:3. Third‐ to fifth‐generation fibroblasts were used for subsequent experiments.

### 2.2 modHGF synthesis and formulation

The modRNAs were synthesized and transfected as previously reported. The pUC57‐HGF/pUC57‐GFP plasmids were constructed according to the HGF/GFP CDS sequence. Clean PCR products generated by plasmid templates were used as templates for mRNA. ModRNAs are generated by in vitro transcription of DNA PCR products from customized ribonucleoside mixtures and plasmid templates, which are subsequently phosphorylated and purified again. The modHGF was dissolved in 10 mM Tris HCl, and 1 mM EDTA at 1 μg/μL for further experiments.

### 2.3 Establishment of modHGF‐modified ADSCs

The transfection of modHGF into ADSCs was performed with MessengerMAX (Invitrogen, California, USA) reagent. The modHGF‐modified ADSCs, namely, HGF‐ADSC.

### 2.4 Wound healing assay

The cells were inoculated on 24‐well plates and cultured to 90% confluency. Subsequently, a straight wound was created at the central axis using a sterile 200 μL pipette. Following rinsing the cells with PBS, the cells were maintained in an FBS‐free medium for a designated duration. Images were captured under a microscope and the wound area was quantified by three independent experiments.

### 2.5 Transwell migration assay

The cells were plated at 5 × 10^4^ cells/100 μL in FBS‐free medium into upper chambers in a transwell insert (Corning, NY, USA). Then, the lower chamber was supplemented with 600 μL of complete medium. Following a 24‐h incubation period, non‐migrating cells were eliminated. The migrating cells were fixed with 4% paraformaldehyde and subjected to crystal violet staining. Visualization of stained cells was performed under a microscope.

### 2.6 Western blot analysis

RIPA lysate was used to lyse cells and the resulting supernatant was collected. The protein concentration was determined by the BCA method. The 30 μg protein sample was added to the prepared 10% polyacrylamide gel, and subjected to electrophoresis. The electrophoretic gel was placed in the membrane transfer instrument at 200 mA for 100 min. After being subjected to a 2‐h sealing process, the sample underwent incubation with diluted primary antibody at 4°C overnight and was then incubated with diluted secondary antibody for 1 h at room temperature. The coloration of the protein band was conducted using an enhanced chemiluminescence substrate kit (Invitrogen, USA).

#### 2.6.1 Enzyme‐linked immunosorbent assay

The concentration of HGF was examined using an ELISA kit (Abcam, USA). The absorbance was determined at 450 nm using a microplate spectrophotometer.

### 2.7 Statistical analysis

Data analysis was conducted using SPSS 22.0. Continuous variables are presented as mean ± standard deviation (SD). Student *t*‐test was utilized to compare the differences between the two groups. One‐way or two‐way ANOVA was employed to examine variation among more than two groups. *p* < 0.05 was considered statistically significant.

## 3 RESULTS

### 3.1 Characterization of human ADSCs

**FIGURE 1:**
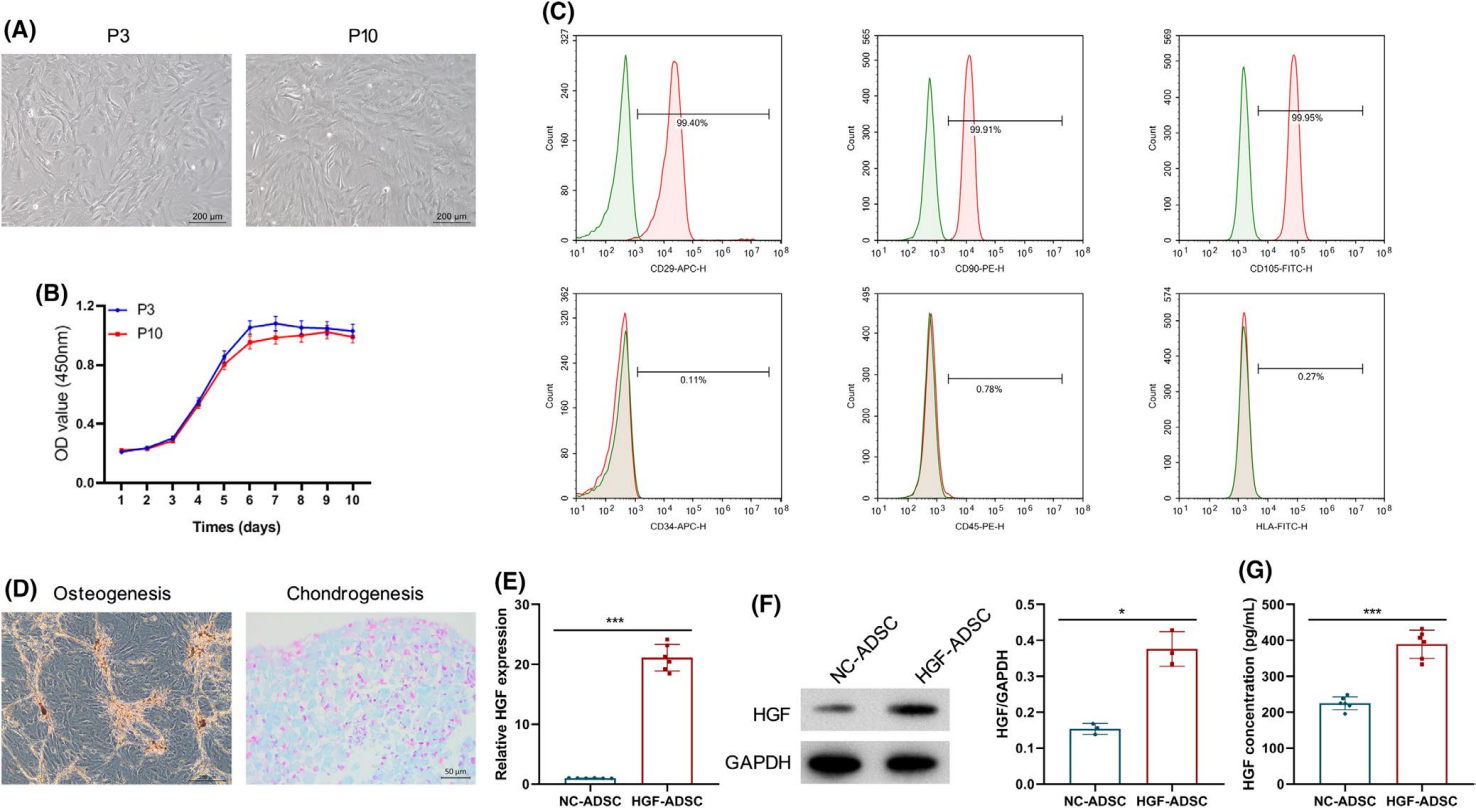
Characterization of ADSCs. (A) Representative images of the third and tenth‐generation ADSCs under an optical microscope. Scale bar = 200 μm. P3: third‐generation ADSC; P10: tenth‐generation ADSC. (B) The growth curve of ADSC of third and tenth‐generation ADSC, determined by CCK8 assay kit. (C). The cell surface markers of ADSCs were measured by flow cytometry. (D) Alizarin red S staining (left) for osteocytes and alcian blue staining(right) for chondrocytes following osteogenic or chondrogenic induction of ADSCs for 3 weeks scale bar = 50 μm. (E) Quantitative real‐time PCR (qRT‐PCR) analysis of upregulated HGF mRNA expression in ADSCs after HGF transfer. (F) Western blot analysis of upregulated HGF protein expression in ADSCs after HGF transfer. (G) The concentration of HGF in the conditioned medium was detected by ELISA assay. *p < 0.05, ***p < 0.001.

The isolated ADSCs were fibroblast‐like, and spindle in shape (Figure 1A). The cell viability of tenth‐generation (P10) ADSCs was slightly lower than that of the third‐generation (P3), demonstrated by the CCK8 assay (Figure 1B). Flow cytometry analysis revealed the positive expression of CD29, CD90, and CD105, and the negative expression of CD34, CD45, and HLA‐DR in ADSCs (Figure 1C), demonstrating that the extracted cells are MSCs. Additionally, positive alizarin red S staining or alcian blue staining was observed after osteogenic or chondrogenic induction of ADSCs for a duration of 3 weeks, supporting the mesenchymal origin of these ADSCs (Figure 1D).

Next, ADSCs were modified with modHGF. As depicted in Figure 1E,F, modHGF modification resulted in an augmentation of HGF mRNA and protein expression in ADSCs. Moreover, the ELISA assay revealed a significantly elevated concentration of HGF in the conditioned medium (CM) of HGF‐ADSC compared to that of NC‐ADSC (Figure 1G). These data indicate that modHGF modification enhances the expression and release of HGF in ADSCs.

### 3.2 Impact of HGF overexpression on the proliferation and migration of ADSCs

**FIGURE 2:**
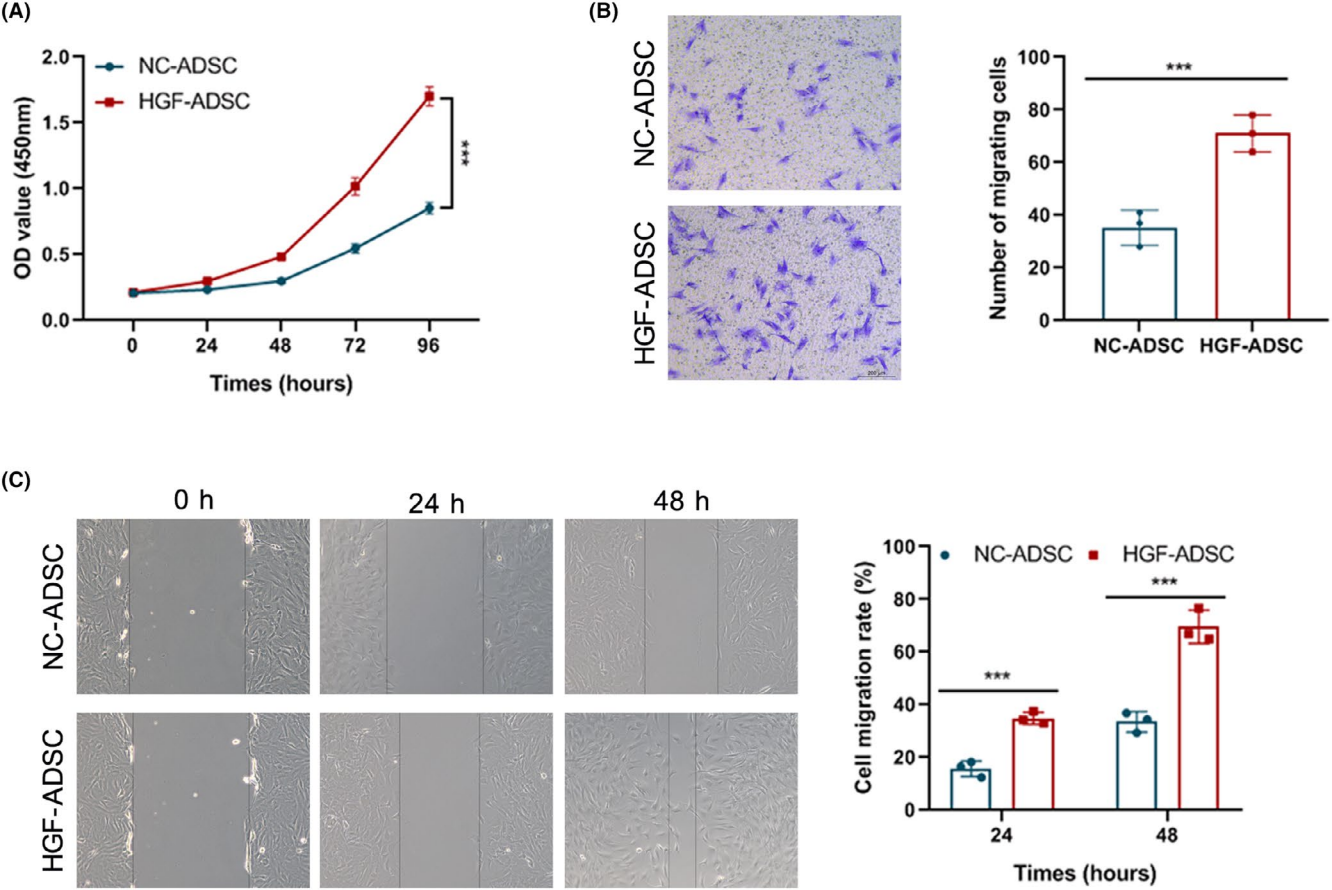
Effect of HGF overexpression on the proliferation and migration capacities of ADSCs. (A) CCK8 assay was performed to analyze the viability of modHGF‐engineered ADSCs. (B) Wound healing assay to evaluate the migration ability of modHGF‐engineered ADSCs. (C) Transwell migration assay was performed to analyze the migration activity of modHGF‐engineered ADSCs. ***p < 0.001 versus the NC‐ADSC groups.

Next, we assessed the effect of modHGF modification on the proliferative and migration abilities of ADSCs. CCK8 assay showed modHGF modification enhanced the cell viability of ADSCs at 48 and 96 h (Figure 2A). Furthermore, transwell migration experiment results show that modHGF modification increased the number of migrating cells (Figure 2B). Additionally, wound healing experiment demonstrated that HGF‐ADSC groups exhibited a significantly accelerated rate of wound healing compared to the NC‐ADSC groups (*p* < 0.01, Figure 2C). These findings suggest that modHGF modification enhances the proliferative and migratory capabilities of ADSCs.

### 3.3 HGF‐ASDCs alleviate the proliferation and migration of HSFs

**FIGURE 3:**
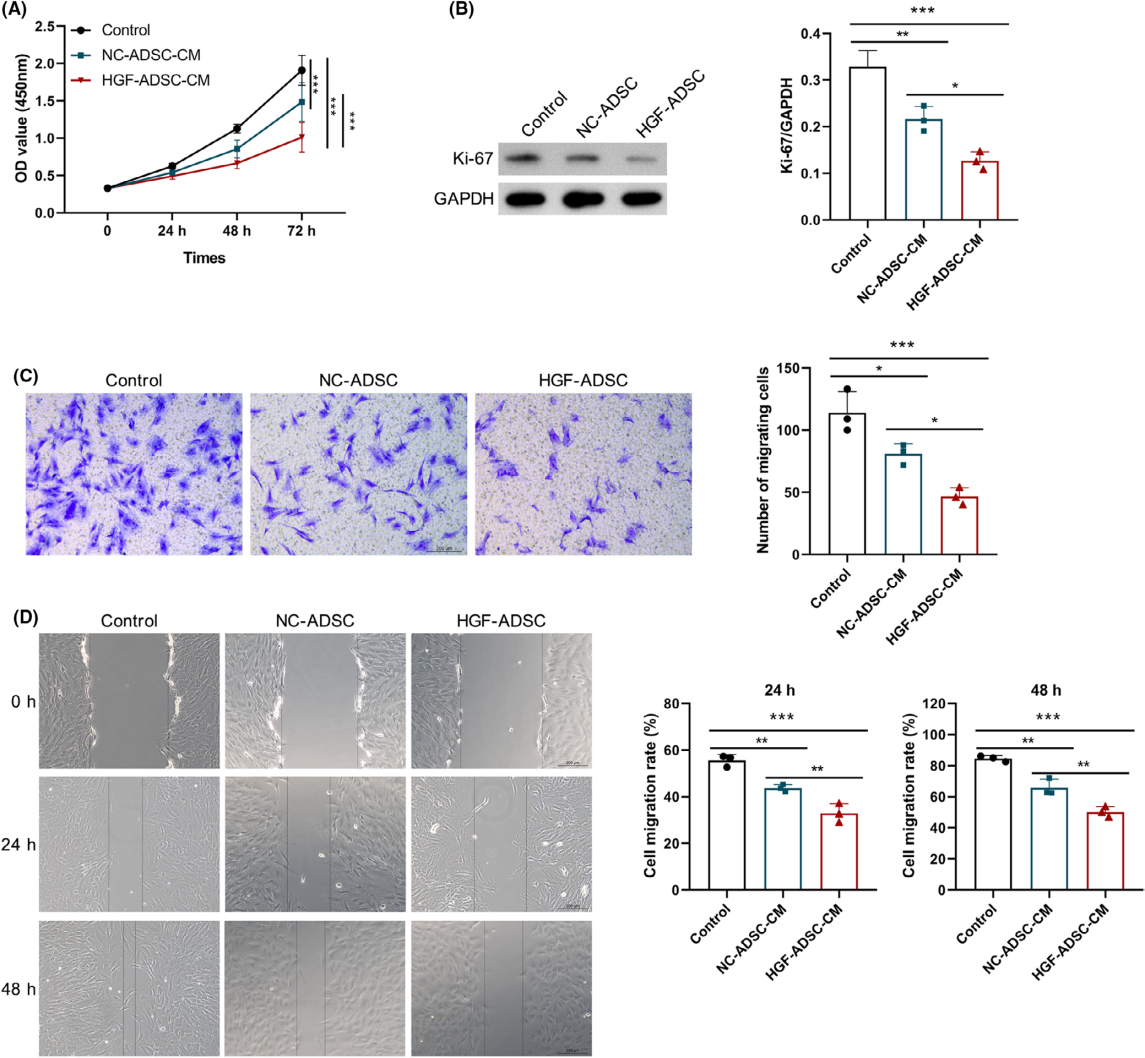
HGF‐ASDCs alleviate the proliferation and migration in HSFs. HSFs were cultured alone, or cocultured with HGF‐ADSC‐CM or NNC‐ADSC‐CM. (A) The viability of HSFs detected by CCK8 assay. (B) The Ki‐67 expression was measured by western blot. (C) Transwell and (D) wound healing assays were performed to analyze the migration ability of HSFs. Scale bar: 200 μm. *p < 0.05, **p < 0.01, ***p < 0.001.

To evaluate the paracrine function of these ADSCs on hHSF cellular function, hHSF were cultured with the culture medium derived from HGF‐ADSC (HGF‐ADSC‐CM) or NC‐ADSC (NC‐ADSC‐CM). CCK‐8 assay revealed that the proliferation rate of hHSFs remarkably increased upon HGF‐ADSC‐CM treatment (Figure 3A). Moreover, the administration of HGF‐ADSC‐CM resulted in a notable enhancement in the expression of Ki‐67, which serves as an indicator for cellular proliferation (Figure 3B). Furthermore, wound healing assay showed that HGF‐ADSC‐CM significantly lowered the migratory ability of HSFs (Figure 3C). Similarly, the result of Transwell assay was consistent with that of the wound healing assay (Figure 3D). These results suggested that modHGF‐engineered ADSCs exhibit superior ability to inhibit HSF cell proliferation and migration.

### 3.4 HGF‐ASDCs mitigate the extracellular matrix deposition of HSFs

**FIGURE 4:**
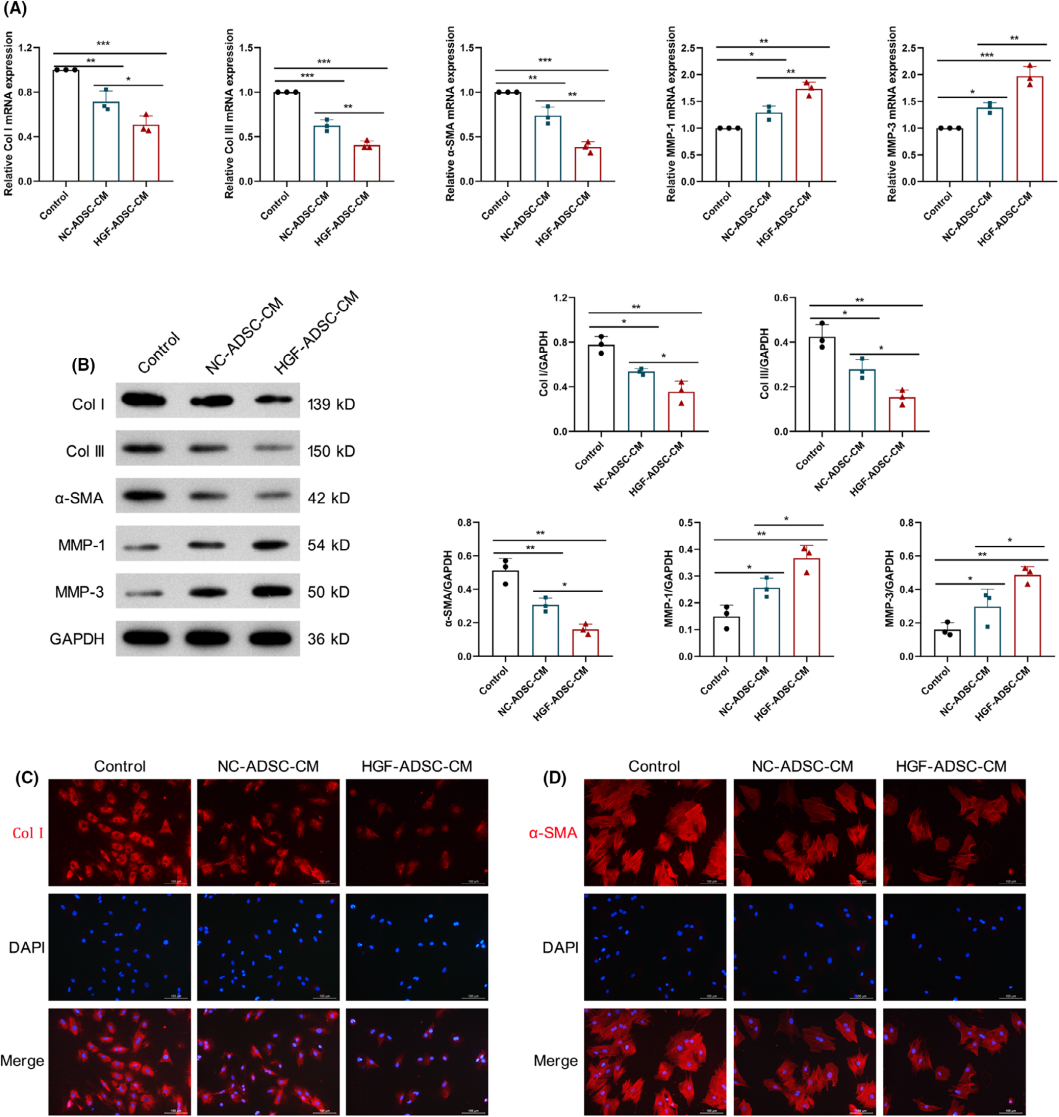
HGF‐ASDCs mitigate the extracellular matrix deposition of HSFs. (A) The expression of collagen‐I (Col I), collagen‐III (Col III), α‐SMA, MMP‐1, and MMP‐3 was measured by QRT‐PCR. (B) The expression of Col I, Col III, α‐SMA, MMP‐1, and MMP‐3 was measured by western blot. (C) The expression of Col I was measured by IF staining. (D) The expression of α‐SMA was assessed by IF staining. Scale bar = 100 μm. *p < 0.05, **p < 0.01, ***p < 0.001.

ECM synthesis and decomposition are also important indicators to evaluate HSF activation. To find out whether modHGF modification affects the ECM synthesis in HSFs, HSFs were cultured alone, or treated with HGF‐ADSC‐CM or NNC‐ADSC‐CM for 3 days, and then, the HSFs were harvested for further analysis. QRT‐PCR showed that the addition of HGF‐ADSC‐CM or NC‐ADSC‐CM significantly reduced the levels of Col I, Col III, and α‐SMA mRNA, and increased the levels of MMP‐1 and MMP‐3 in HSFs. Notably, the effect of HGF‐ADSC‐CM on these genes was greater than that of NC‐ADSC‐CM (Figure 4A). Similarly, when we added HGF‐ADSC‐CM or NC‐ADSC‐CM to the HSFs culture system, the expression of Col I, Col III, and α‐SMA protein in HSFs was significantly decreased, and the expression of MMP‐1 and MMP‐3 in HSFs was significantly elevated. And, the impact of HGF‐ADSC‐CM on these proteins was greater than that of NC‐ADSC‐CM (Figure 4B). To visually observe the differences in the expression of these proteins, Col I and α‐SMA were detected using IF staining. As depicted in Figure 4C‐D, the fluorescence intensity of Col I and α‐SMA was lowest in HSFs treated with HGF‐ADSC‐CM. This finding indicates that modHGF could enhance the inhibition of ADSCs on the synthesis of ECM.

### 3.5 Neutralization of HGF eliminates the suppressive effect of HGF‐ASDCs on HSFs

**FIGURE 5:**
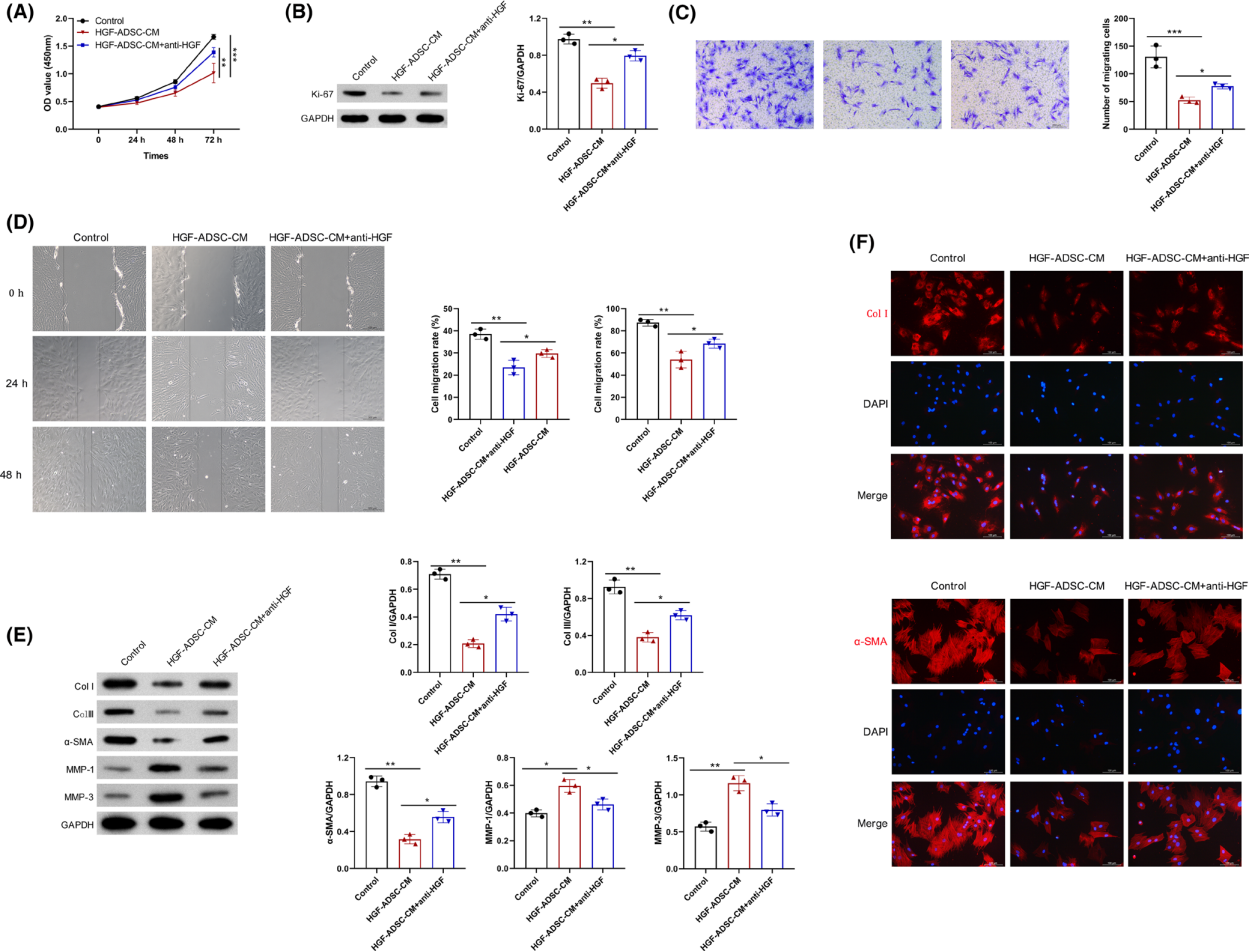
Neutralization of HGF by HGF‐Ab. (A) HSFs were treated with HGF‐ADSC‐CM alone or in combination with human IgG or HGF‐Ab, and cell viability was evaluated by CCK‐8. (B) Western blot analysis of Ki‐67 in HGF‐ADSC‐CM‐treated HSFs with IgG or HGF‐Ab. Wound healing assay (C) and Transwell assay (D) in HGF‐ADSC‐CM‐treated HSFs with IgG or HGF‐Ab. Scale bar: 200 μm. (E) Western blot analysis of collagen‐I, collagen‐III, α‐SMA, MMP‐1, and MMP‐3 in HGF‐ADSC‐CM‐treated HSFs with IgG or HGF‐Ab. (F) Immunofluorescence staining of Col I and α‐SMA in HSFs treated with vehicle, HGF‐ADSC‐CM, or HGF‐Ab. Scale bar: 100 μm. *p < 0.05, **p < 0.01, ***p < 0.001.

To further validate the impact of HGF on hHSF activation, hHSFs were treated with a humanized neutralizing antibody targeting HGF while being incubated with HGF‐ADSC‐CM. Compared to control group, an obvious increase in cell proliferation and Ki‐67 expression was observed in the hHSFs. However, this effect was attenuated when treated with HGF‐Ab (Figure 5A,B). Moreover, treatment with HGF‐ADSC‐CM resulted in decreased migration capacity compared to the control group, as evidenced by lower wound healing rate and reduced number of cells passing through the transwell chambers. While neutralization of HGF using HGF‐Ab weakened the inhibitory effect of HGF‐ADSC‐CM on cell migration (Figure 5C,D). In addition, downregulated Col I, Col III, and α‐SMA proteins along with upregulated MMP‐1 and MMP‐3 proteins were observed in hHSFs treated with HGF‐ADSC‐CM. However, these impacts were significantly reversed upon treatment with HGF‐Ab (Figure 5E). Furthermore, immunofluorescence staining revealed a significant decreased in fluorescence intensity of Col I and α‐SMA in HSFs treated with HGF‐modified ADSC‐CM. Strikingly, HGF‐Ab treatment reversed the decrease of Col I and α‐SMA (Figure 5F). In combination, these results indicate that HGF‐modified ADSCs effectively inhibited the activation of hHSFs.

## 4 DISCUSSION

The formation of HSs is the inevitable consequence of aberrant wound repair following severe skin trauma, burns, or infection. Due to the unclear underlying pathological mechanism associated with HS, current preventive and therapeutic approaches have not yielded satisfactory outcomes. Numerous clinical and basic studies have substantiated the ability of ADSCs to impede scar formation and enhance wound healing. However, due to low biological activity and insufficient maintenance transitory of ADSCs, their performance and therapeutic effects need to be further optimized. ADSCs can generate and secrete various biologically active components including cytokines, external vesicles, exosomes, DNA, and RNA. Recent research findings indicate that these biologically active substances are the vital pathways by which ADSCs exert biological functions,^21^ such as enhancing tissue repair and impeding fibrosis.^22^, ^23^ In this study, we combined ADSCs with modHGF modification to improve their performance and therapeutic effects. As expected, modHGF modification not only promoted the proliferation and migration of ADSCs but more importantly, it remarkably enhanced the inhibitory effect on the activation and ECM synthesis of HSFs via promoting the release of HGF.

HGF is a multifunctional cytokine, that can exert diverse biological effects depending upon the cell type.^24^, ^25^, ^26^ Previous studies have demonstrated that HGF possesses proliferative and pro‐motility properties, induces survival and growth of epithelial and endothelial cells, and promotes migration of ADSCs, ultimately facilitating tissue repair.^27^ For instance, Zhang J et al.^28^ reported that HGF enhances ADSC migration toward damaged tissue sites while inhibiting hepatocyte apoptosis. In line with these reports, our study revealed a significant enhancement in the viability and migration capacity of HGF‐modified ADSC cells. However, HGF does not confer benefits on fibroblast growth and can inhibit fibroblast activation toward the myofibroblast phenotype. Jiang D et al.^29^ revealed that HGF could suppress the release of collagen and alpha‐SMA from healing fibroblasts. Wang M et al.^20^ demonstrated that HGF prevents fibrosis through interacting with c‐Met. Thus, HGF has been considered as a candidate for scarring improvement therapy. In this study, HGF was transfected into ADSCs by modRNA technology to improve HGF expression and bioactivity in conditioned media. Our data showed that the CM from ADSCs could inhibit the proliferative, migratory abilities and ECM synthesis of hHSFs, while modHGF significantly enhanced these effects. These findings suggest that modHGF could serve as a potential therapeutic tool for the performance and therapeutic effects of ADSCs.

Our study had several limitations. First, we did not validate the therapeutic efficacy of HGF‐modified ASDCs in improving scar formation in vivo experiments. Second, further exploration is needed to understand how HGF‐ASDCs exert their effects at different stages of treatment and whether there is an optimal time window for achieving enhanced therapeutic outcomes with HGF‐ASDCs. These aspects may hold the key to preventing and reversing HSs.

## 5 CONCLUSION

In summary, our data show that modHGF‐engineered ADSCs show an excellent ability to inhibit activation and ECM synthesis of HSFs, suggesting the potential to prevent scarring.

## AUTHOR CONTRIBUTIONS

T.Z. prepared Figures 1, 2, 3, 4, 5. T.Z. wrote and reviewed the main manuscript text.

## FUNDING INFORMATION

None.

## CONFLICT OF INTEREST STATEMENT

The authors declare no potential conflict of interest.

## ETHICS APPROVAL

This study was approved by the Ethics committee of Wuhan University of Science and Technology (No. 20210415).

## Supporting information


Appendix S1.

## Data Availability

The data that supports the findings of this study are available in the supplementary material of this article (Appendix S1).
