# Supplementary material for: Hepatocyte growth factor‐modified adipose‐derived mesenchymal stem cells inhibit human hypertrophic scar fibroblast activation
Source: J Cosmet Dermatol. 2024 Aug 18;23(12):4268–76. doi: 10.1111/jocd.16509 (PMC11626330; doi:10.1111/jocd.16509)

Figure 1F

HGF

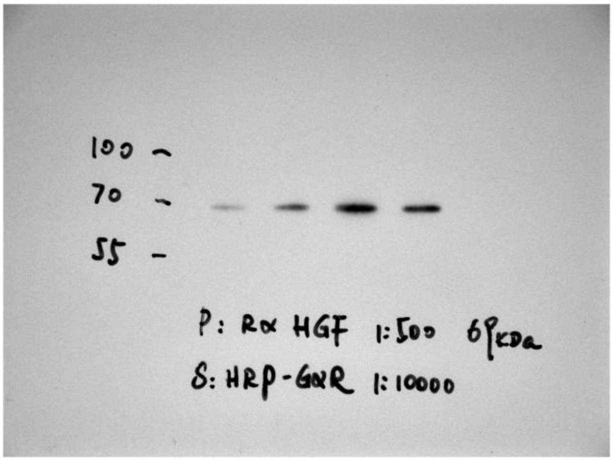

GAPDH

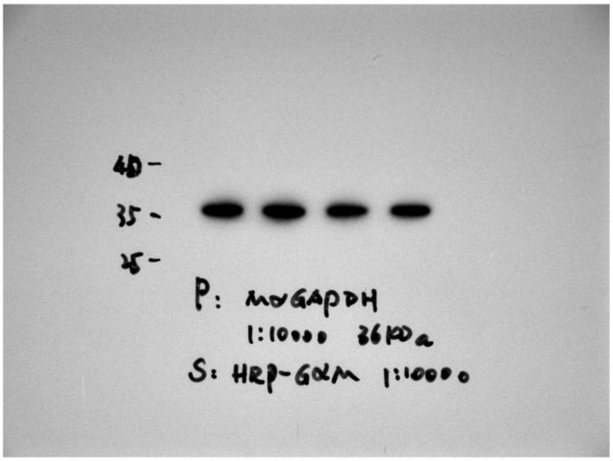

Figure 3B

Ki-67

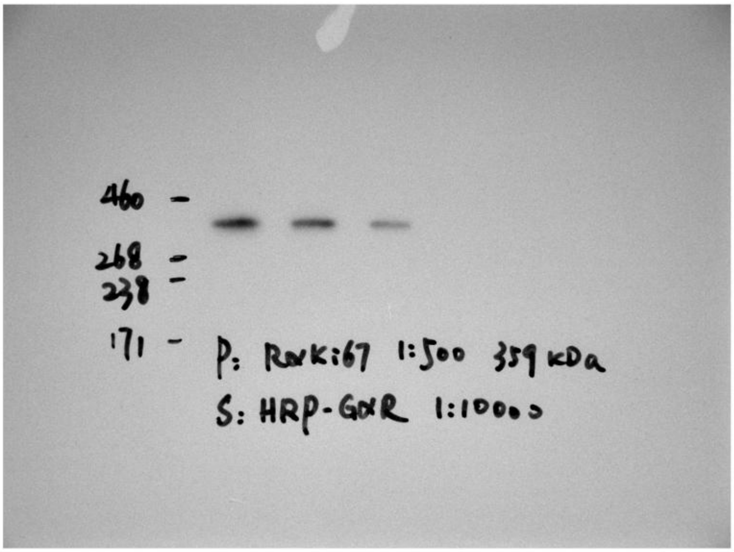

GAPDH

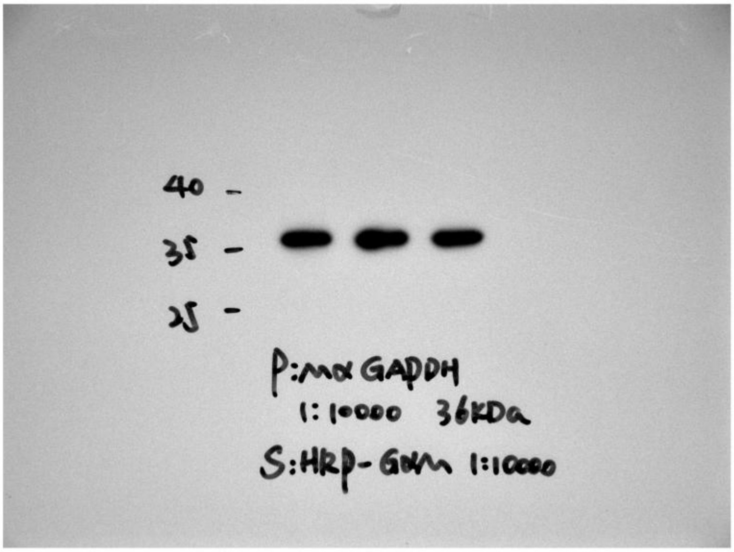

Figure 4B-1

Col I

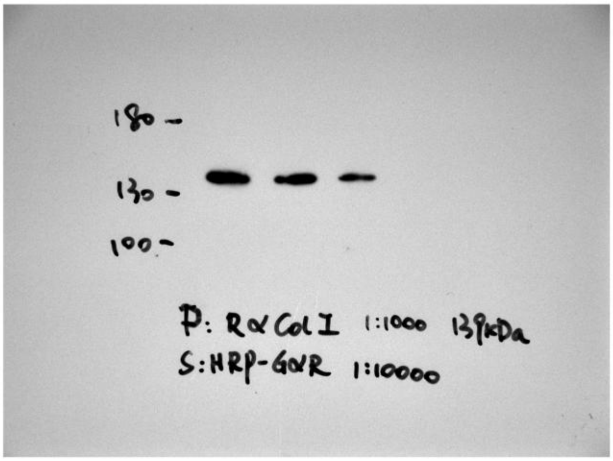

Col III

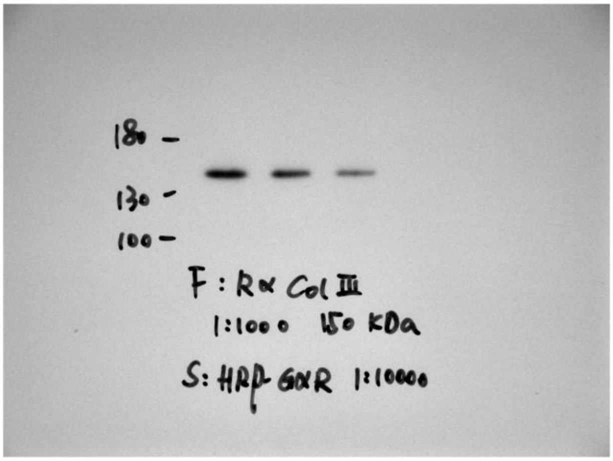

α-SMA

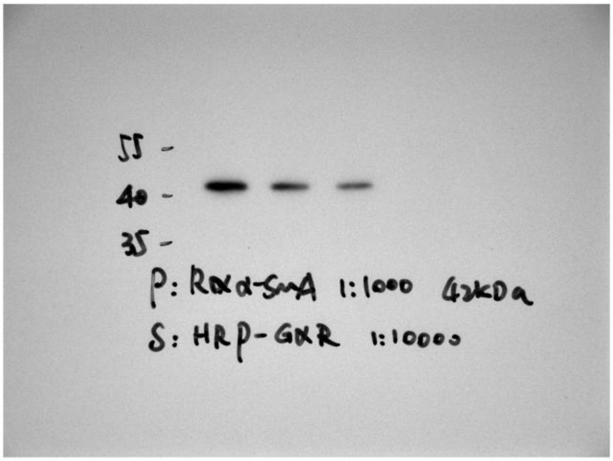

Figure 4B-2

MMP-1

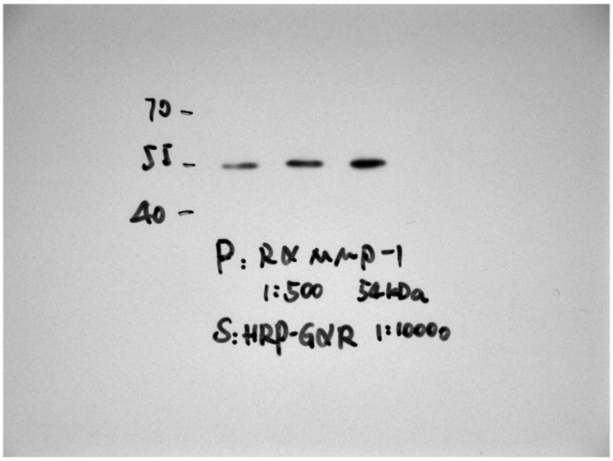

MMP-3

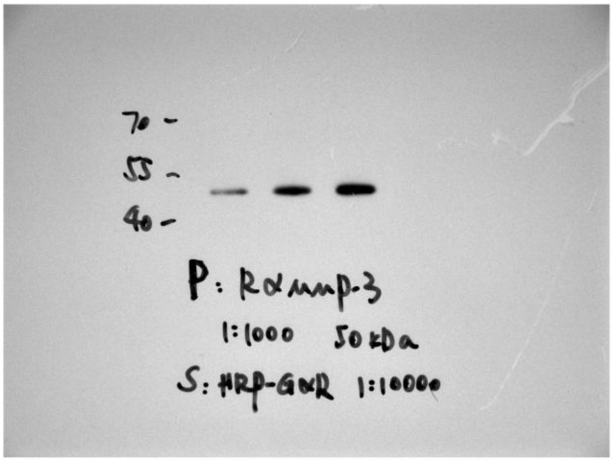

GAPDH

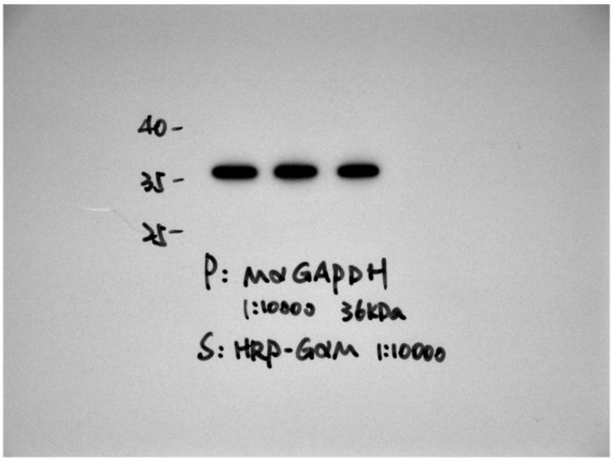

Figure 5B

Ki-67

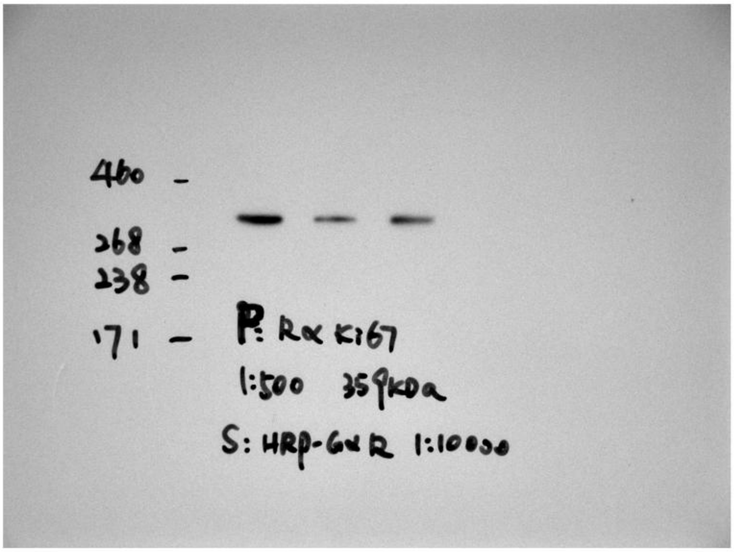

GAPDH

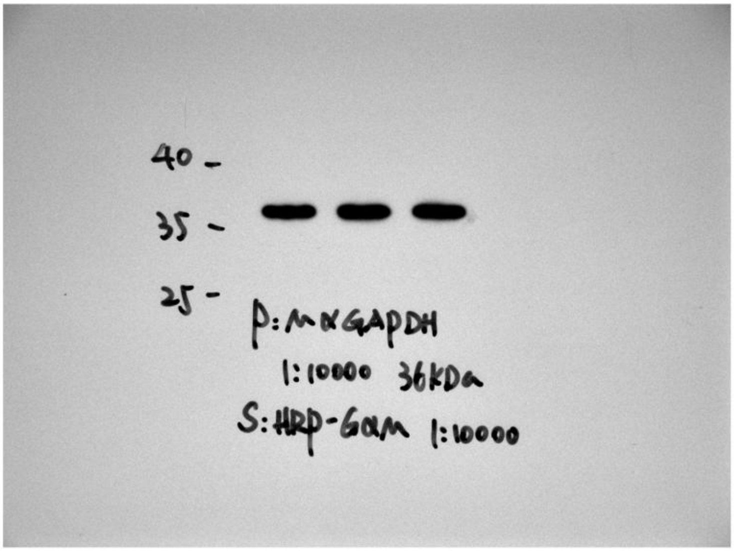

Figure 5E-1

Col I

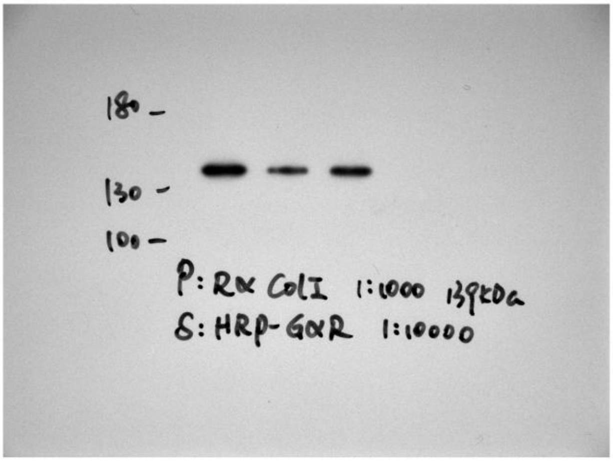

Col III

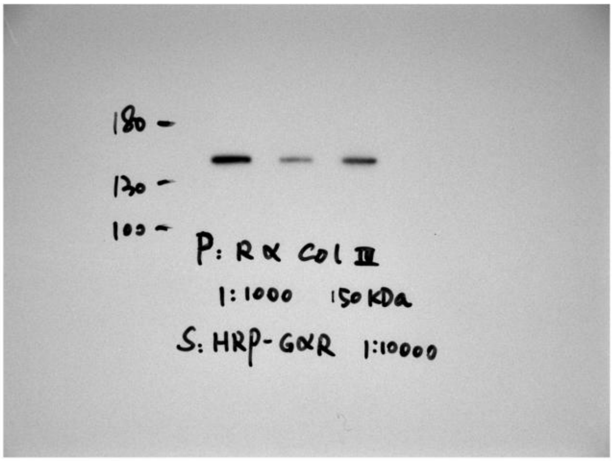

α-SMA

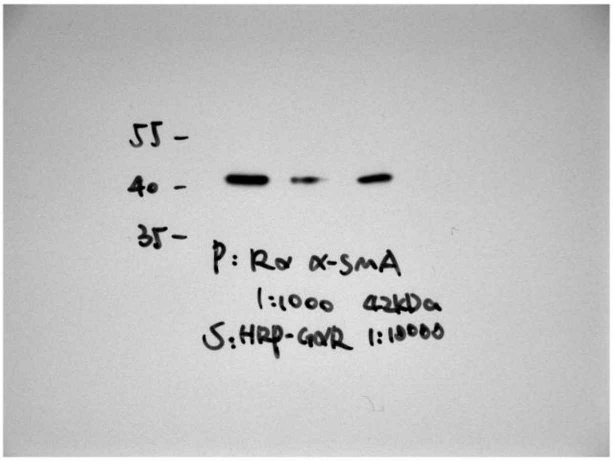

Figure 5E-2

MMP-1

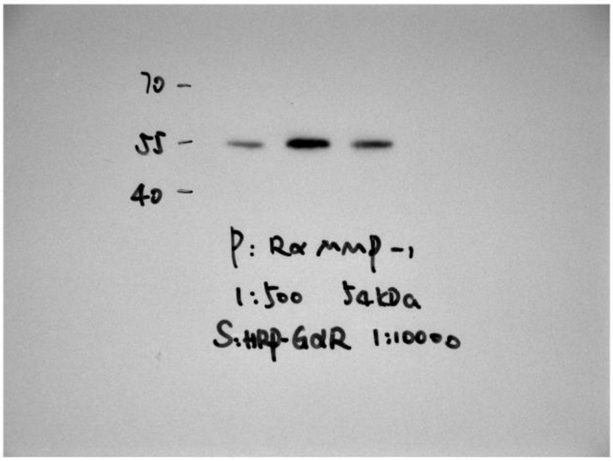

MMP-3

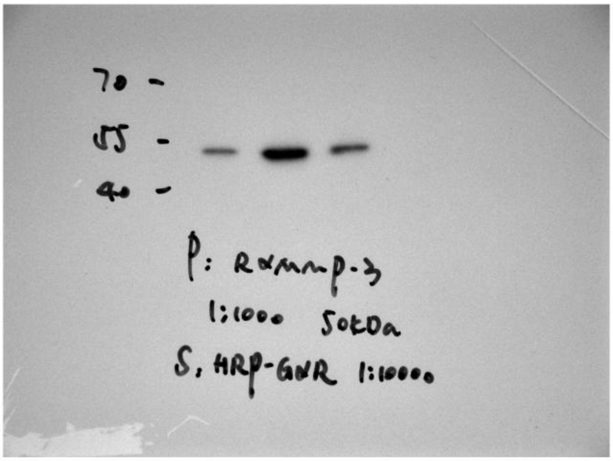

GAPDH

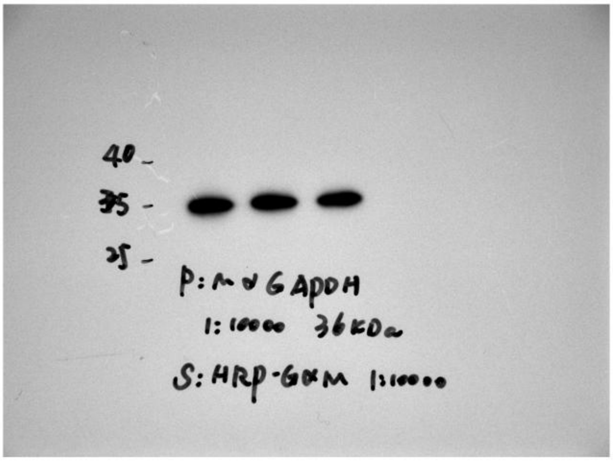

Supplement: Supplementary file 1 — Appendix S1. [file JOCD-23--s001.pdf]
